# The Anophthalmic Socket – Reconstruction Options

**Published:** 2014

**Authors:** S Schmitzer, C Simionescu, C Alexandrescu, M Burcea

**Affiliations:** * Emergency Eye Hospital, Bucharest; ** Oftalmestet Clinic, Bucharest; *** Oftalmestet Clinic, Bucharest

**Keywords:** anophthalmia, reconstruction, reconstruction

## Abstract

Keeping the eye is impossible, functionality is lost and aesthetic requirements are high. What do we do? Which approach is right? This is the dilemma we face whenenucleation or evisceration are unavoidable. The patient loses a sensory organ with a very important function and, at the same time, is faced with a major aesthetic defect, leading to significant anxiety. The purpose of this article is to describe the different reconstruction techniques for anophthalmic sockets. In preparing the anopthalmic socket for prosthesis fitting we have several options: dermis-fat graft with a very good biocompatibility, the Guthoff artificial implant, which provides better motility or the methyl methacrylate implant inside the muscle cone. Each option has advantages and disadvantages but the choice of technique should be adapted to the needs and expectations of the patient, taking into account both time and cost.

## Introduction

There are situations when, in order to alleviate severe pain, to protect a healthy contralateral eye, to improve aesthetics or even to save the life of a patient, the eyeball must be removed. Although this approach solves the patient's health problem, by changes in physiognomy it can have a devastating effect on their perception and self-confidence. In front of a patient in whom visual function cannot be recovered the doctor should take all measures to offer the patient a “normal” look normal after surgery. [**1**] The patient wants the ideal ocular prosthesis, natural looking, that friends and family don’t know about. From the medical point of view this means eyelids in normal position, the ability to blink, eyelashes in normal position, symmetrical with the healthy eye, good motility, custom prosthesis. When dealing with an anophthalmic socket, the surgeon can help patients by restoring the volume lost by removing the eyeball and by restoring orbital architecture. Orbital volume can be restored with the help of. We will discuss below some types of orbital implants used after evisceration or enucleation. Exenteration, partial or total, has completely different reconstruction techniques that will not be addressed in this article.

**Orbital implants**

Once the eyeball removed, with care and minimizing the alterations to the orbital content, attention is focused on restoring the orbit volume. The volume of an enucleated eyeball varies between 7 and 9 ml, with a mean of 7.9 ml. [**2**] The deficit volume after enucleation or evisceration is corrected by an orbital implant and an ocular prosthesis. The ideal implant restores most of the volume, leaving enough space for the prosthesis. The latter only restores a maximum of 4.2 ml of the volume required. [**3**] Too large orbital implants have increased risk of expulsion and leave little room for prosthesis. That is why the prosthesis will be too thin to create the impression of a deep anterior chamber. On the other hand, a too small orbital implant will not restore lost volume leading to enophthalmos and deepening of the upper eyelid sulcus. In this case the prosthesis should be larger but this cause discomfort and reduced motility. In a retrospective study Katreider evaluated the volume replacement afterenucleation by the orbital implant and prosthesis. He determined the volume of the healthy eye and assumed the enucleated eyeball has the same volume. The upper eyelid sulcus deepening and enophthalmos were more common in patients whose orbital volume replacement (implant and prosthesis) was smaller than the volume of the healthy eyeball. According to his results, it is recommended to perform an ultrasound A-scan of the healthy eye to assess the necessary volume of the orbital implant. [**4**]. There are several types of orbital implants, depending on the time of placement (primary or secondary), depending on the source (natural or artificial), and depending on the material (porous or non-porous). Choosing the appropriate implant depends on several criteria such as patient age, history or availability of implant, surgeon’s preference and cost. In literature the ideal orbital implant is described as: chemically and biologically inert, easily inserted without sharp edges, easily covered with conjunctiva above, low risk of expulsion or migration, able to provide better motility and, last but not least, low cost. [**5**]

The orbital implants we use in current practice are methyl methacrylate sphere, hydroxyapatite implant, Guthoff orbital implant, dermis-fat graft and conformer dressed in skin graft.

**Methyl methacrylate sphere**

The methyl methacrylate sphere is part of the non-porous orbital implants and is most commonly used to restore volume lost after evisceration. In this case the implant is placed in the scleral bag, ensuring adequate good volume and motility. (**Fig. 1a**,**1b**) Even if the implant is placed in the scleral bag and sclera sutured over, there is a risk of expulsion if the implant is too large, the sutures are loose or the conjunctival suture is performed in a single plane. In our practice we found that it is more efficient in the long term to perform duplicationof conjunctiva and Tenon's capsule and to suturein two layers. However,between the two layers serous cysts can develop leading to the displacement of the prosthesis but these can beeasily excised. (**Fig. 2**)

**Fig. 1a:**
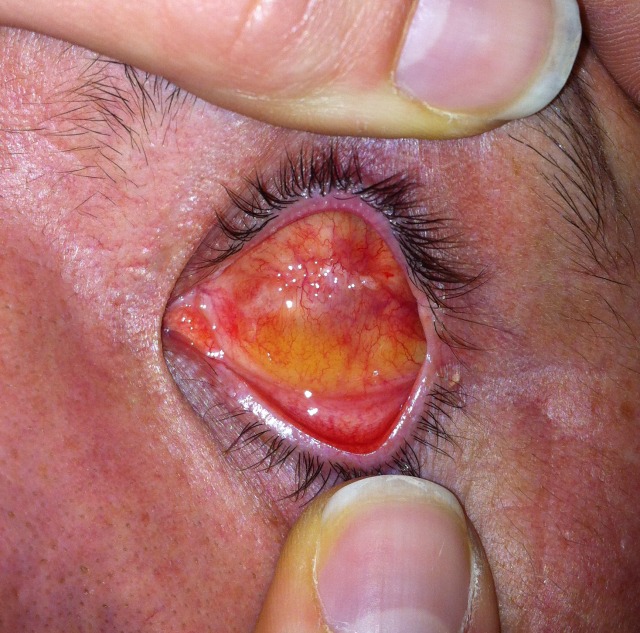
Evisceration with methylmetacrylate sphere

**Fig. 1b:**
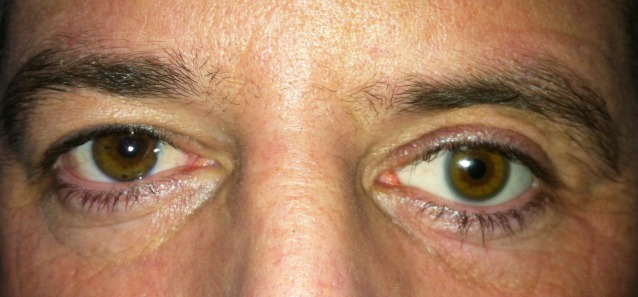
Methylmetacrylate sphere and prosthesis

**Fig. 2:**
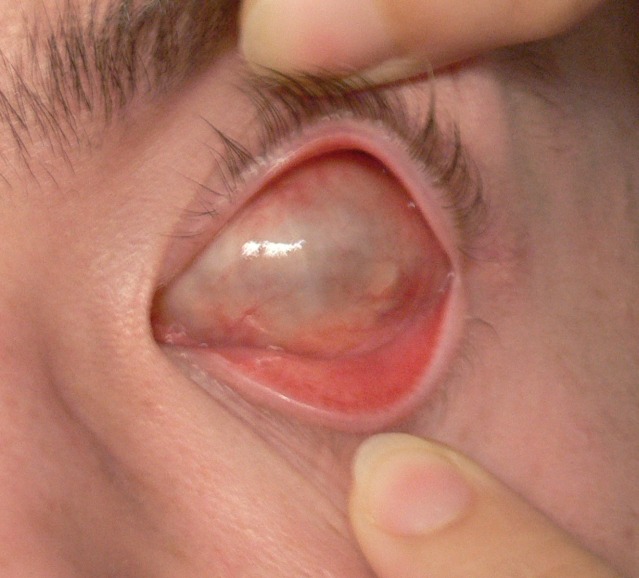
Serous cyst between Tenon’s capsule and conjunctiva

The implant can also be placed within the muscle cone, behind the scleral bag. You make a posterior sclerotomy and, after placing the implant in the muscle cone, sclera and conjunctiva are sutured above. In this situation the risk of expulsion diminishes and it provides good volume with good motility. (**Fig. 3a**, **3b**) This technique can be performed per primam, if the scleral bag is small, shrunken (atrophic globe) and does not allow placing the implant inside or per secundam, years away after evisceration, when implant expulsion occurs. 

**Fig. 3a:**
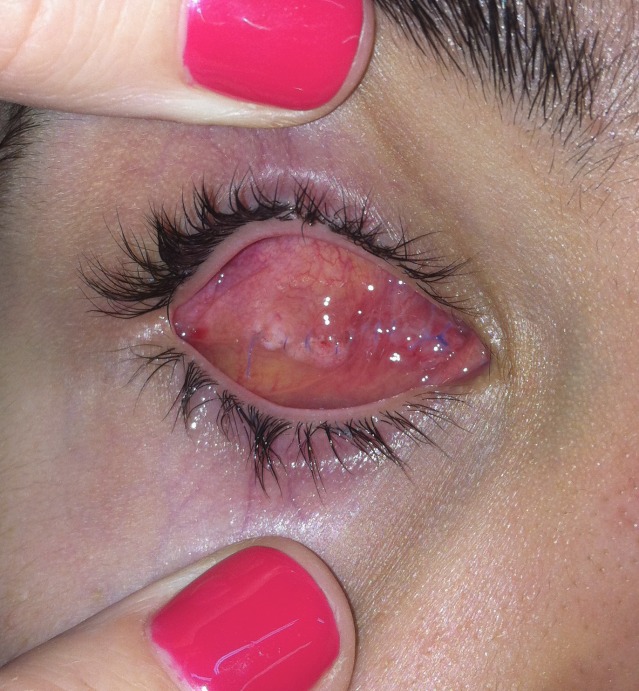
Methylmetacrylate sphere placed within the muscle cone

**Fig. 3b:**
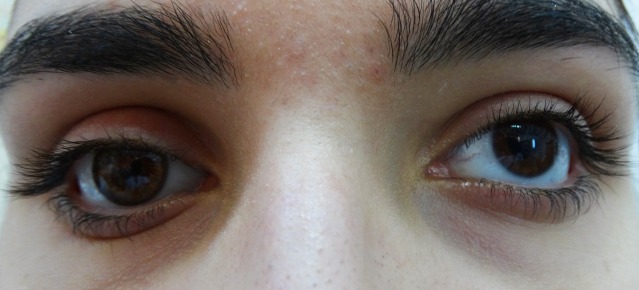
Methylmetacrylate sphere in the muscle cone and prosthesis

**Fig. 4:**
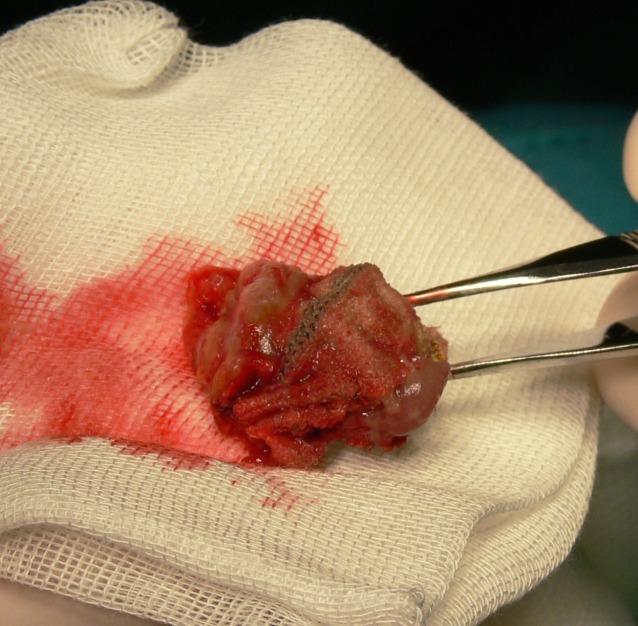
Methylmetacrylate implant wrapped in mesh

This type of implant may be used after enucleation if it is wrapped in a porous material (Teflon) or sclera harvested from cadavers. Both options require extraocular muscle integrity to ensure adequate motility. Unfortunately, both techniques have disadvantages. The implant wrapped in Teflon has a high risk of expulsion and infection (**Fig. 4**) and implant wrapped in sclera harvested from cadavers runs the risk of transmitting viral or prion disease. [**6**] It is not recommended to place methyl methacrylate implant directly into the orbital cavity after enucleation because, since the extraocular muscles are not connected, there is a risk of expulsion or migration, most commonly in the superotemporal space [**7**]

**Hydroxyapatite implant**

Used in enucleation and sometimes in evisceration, the hydroxyapatite implant was discovered in 1985 by Perry, obtaining approval in 1989. (8) Originally obtained from coral, biocompatible, it represented a new generation of integrated orbital implants with a network of interconnected pores to form a fibrovascular mesh within the orbit (8). As such, it was considered that this diminishes the risk of expulsion and, by suturing the extraocular muscles, it allows a very good motility of the prosthesis. Another considerable advantage at the time was that hydroxyapatite implant allows placing a peg that connects to the prosthesis, offering a very good motility. It was later found that this pegging technique involves multiple risks of infection and expulsion and it is now contraindicated in elderly patients, immunosuppressed or after local radiotherapy.

**Fig. 5:**
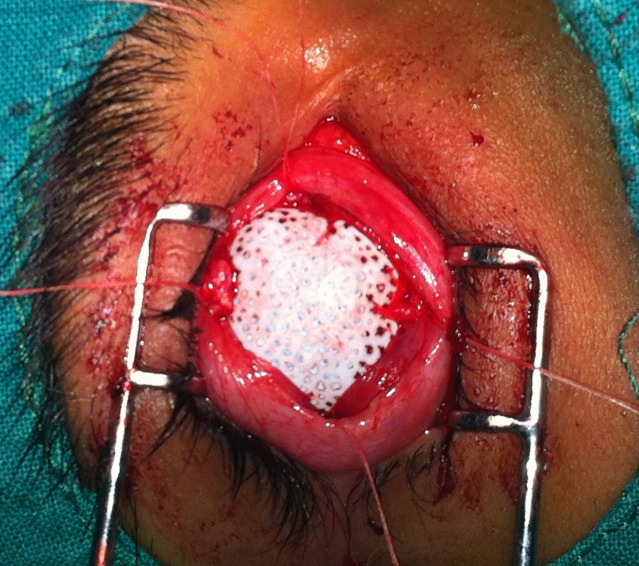
Hydroxyapatite implant

Although it was an innovation at the time of discovery, over 20 years of surgeon’s experience of surgeons revealed multiple complications such as expulsion, conjunctival thinning, infection, orbital pain in the long term [**9**, **10**, **12**] Especially the risk of expulsion makes this type of implant to be used less and less, although this complication is related more to the surgical technique than to the actual implant. (**Fig. 5**) [**11**, **13**, **14**] Also, discovering this implant greatly increased the cost of surgery, the implant being expensive compared with the methyl methacrylate sphere, for example.

**Guthoff orbital implant**

**Fig. 6:**
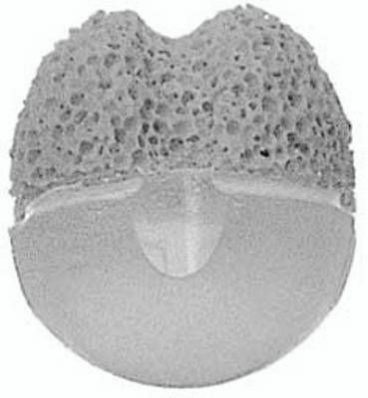
Guthoff implant

**Fig. 7:**
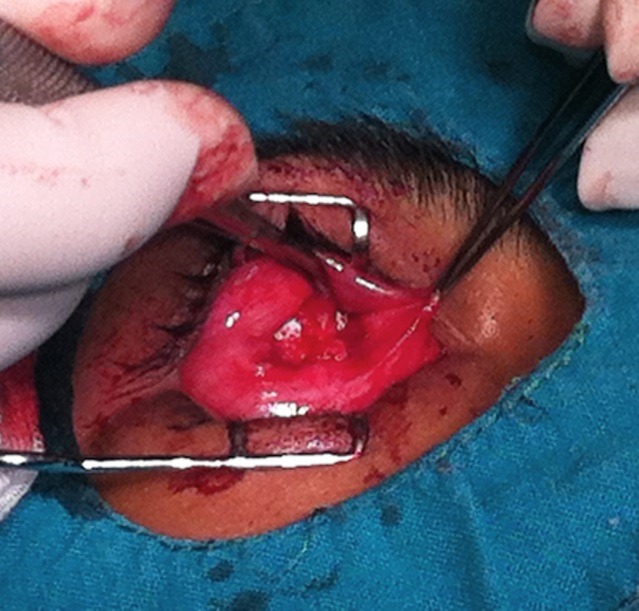
Guthoff implant - surgery

Invented by Professor R. Guthoff a few years ago, it combines the advantages of the hydroxyapatite implant with the ones of methyl methacrylate sphere. It is divided into two areas; the posterior half is methyl methacrylate and anterior half of hydroxyapatite, where there are four grooves for suturing the extraocular muscles (**Fig. 6**). It offers good orbital volume, very good motility and very good integration. Unfortunately this orbital implant is an expensive one and it is necessary to have available several sizes during surgery. (**Fig. 7**) Also, being quite recently emerged, further studies are needed to assess efficacy in the long term.

**Dermis fat graft**

**Fig. 8:**
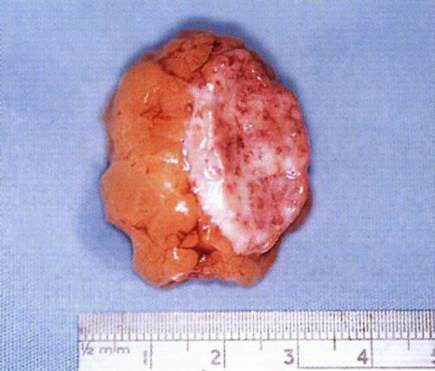
the dermis fat graft

Implantation of autologous material in the anophthalmicsocket reduces the risk of migration or expulsion present in artificial implants. The dermis-fat graft, with perfect biocompatibility, has two components: fat to restore orbital volume and dermis that provides vascular support for the graft (the dermis takes the epithelial characteristics of the environment where it is implanted, not of the tissue of origin) (**Fig. 8**) The dermis fat graft has multiple indications: congenital anophthalmia, contracted anophthalmic socket (if there is enough vascularized tissue), after expulsion of an artificial orbital implant, in enucleation per primam or per secundam. Enucleation should be done preserving as much of the vascular support required for graft integration, especially conjunctiva, Tenon’s capsule and extraocular muscles. Socket dissection should be minimal to reduce the risk of fibrosis and not to undermine future graft vascularization. [**15**]

The dermis fat graft is harvested from the hip or groin area, in an area without hair that can be easily covered by a bathing suit and not located in a region of pressure. It is also necessary to check if the patient has enough fat panicle and has no skin infections. The anterior surface of the graft should have a diameter between 20 and 24 mm and a thickness of about 20 mm. Small grafts lead to under correction and large grafts run the risk of central necrosis due to compression and ischemia. [**16**]]

Its advantages are manifold (**Fig. 9a**, **9b**) - perfect biocompatibility, allows deepening of the conjunctival fornixes, allows local radiotherapy (in enucleation for intraocular tumors) and patients do not experience any pain or discomfort in the long term. There are some drawbacks, most notably the tendency to atrophy. This process is physiological, but can be accelerated if the graft is injured or poorly vascularized. For this reason as much ofthe vascular support should be preserved,trauma to the socket reduced and adapts the graft volume to the socket. Graft vascularization can be checked from the first postoperative day by the appearance of small blood stains on the surface of the dermis. In the best operating conditions, estimated 25-35% atrophyoccurs in the first 6 months. Atrophy is greater if the socket has been traumatized, irradiated, infected or scarred.

**Fig. 9a:**
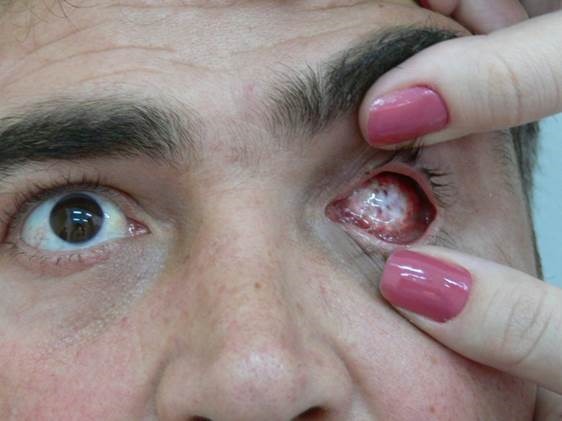
The dermis fat graft – 3 days after surgery

**Fig. 9b:**
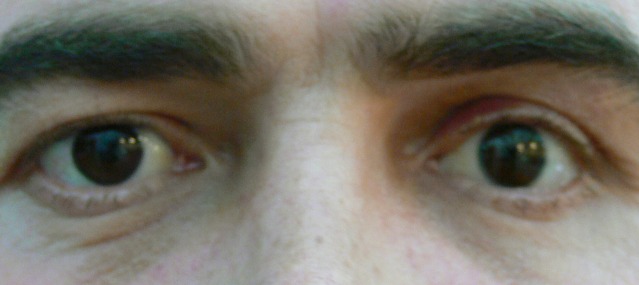
The dermis fat graft and prosthesis ( left eye)

**Conformer enveloped in skin graft**

**Fig. 10:**
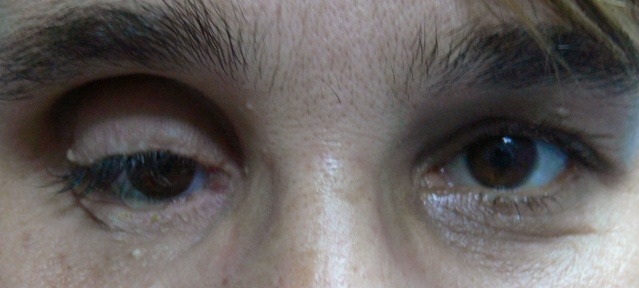
Conformer enveloped in skin graft, hyaluronic acid lower sulcus

In case we are dealing with a severely contracted socket where extraocular muscles cannot be recovered there is the option of placing a conformer wrapped in a skin graft within the socket to restore the orbital volume. The conformer can be biconvex, when orbital volume is diminished or convex-concave if the volume is acceptable but the conjunctival fornixes are retracted. The skin graft is harvested from the inner side of the arm, in an area without hair and the conformer is enveloped, placing the epidermis in in contact with the conformer. This technique represents the ultimate solution for reconstruction, when no other option will work. Therefore the disadvantages are mostly aesthetic with no prosthesis motility and difficulties in eyelid closure. Postoperatively, inorder improve orbital volume and upper and lower eyelid grooves, hyaluronic acid can be injected into the socket or a fat graft may be used to restore volume of the cavity and of the sulcus, providing a satisfactory aesthetic result. (**Fig. 10**)

## Conclusions

There are multiple types of orbital implants, each with advantages and disadvantages. It isimportant to keep in mind the criteria for choosing an implant, one of the most important being the surgeon’s experience. In our experience, the best results we have obtained with the dermis fat graft or the Guthoff implant, the only criterion to differentiate between the two is, at present, the cost.

Loss of one eye due to a tumor, trauma or eye disease is devastating for the patient at any age. The patient loses binocular vision with reduced visual field but suffers also changes in the perception of self that can lead to anxiety and depression. Eye contact is an important part of interpersonal interaction and thus is essential for the patient to receive a more natural prosthesis. Anophthalmic socket surgery is no longer just the replacement of a diseased eye with an orbital implant. The collaboration between surgeon and ocularist is essential in order to provide a natural-looking prosthesis with good motility.
